# Parallels between vertebrate cardiac and cutaneous wound healing and regeneration

**DOI:** 10.1038/s41536-018-0059-y

**Published:** 2018-11-07

**Authors:** Rebecca J. Richardson

**Affiliations:** School of Physiology, Pharmacology and Neuroscience, Faculty of Biomedical Sciences, University of Bristol, Bristol, UK

## Abstract

The cellular events that contribute to tissue healing of non-sterile wounds to the skin and ischaemic injury to internal organs such as the heart share remarkable similarities despite the differences between these injury types and organs. In adult vertebrates, both injuries are characterised by a complex series of overlapping events involving multiple different cell types and cellular interactions. In adult mammals both tissue-healing processes ultimately lead to the permanent formation of a fibrotic, collagenous scar, which can have varying effects on tissue function depending on the site and magnitude of damage. Extensive scarring in the heart as a result of a severe myocardial infarction contributes to ventricular dysfunction and the progression of heart failure. Some vertebrates such as adult zebrafish, however, retain a more embryonic capacity for scar-free tissue regeneration in many tissues including the skin and heart. In this review, the similarities and differences between these different types of wound healing are discussed, with special attention on recent advances in regenerative, non-scarring vertebrate models such as the zebrafish.

## Introduction

Vertebrates have a remarkable capacity for healing traumatic injuries to the majority of tissues and organs. Wound healing in the skin, for example, can repair serious and extensive surgical and traumatic injuries even in a non-sterile environment. Similarly, serious ischaemic injury, where occlusion of blood vessels results in a region of tissue experiencing a transient loss of oxygen within internal organs such as the heart, can be effectively repaired. The long-term consequences of these effective healing regimes can, however, be further detrimental to the function of these tissues and has varying effects on organ function. Although our ability to repair serious tissue trauma remains relatively remarkable, mammals lose any significant ability to regenerate lost cells and tissues or to heal in a scar-free fashion during early post-natal periods.^1–3^ Non-mammalian adult vertebrate models such as zebrafish, axolotls and newts, however, retain more embryonic capacities to regenerate lost cells, replace regions of tissue and to resolve deposited scar tissue to heal in a perfect, scar-free fashion.^4–9^

**Fig. 1:**
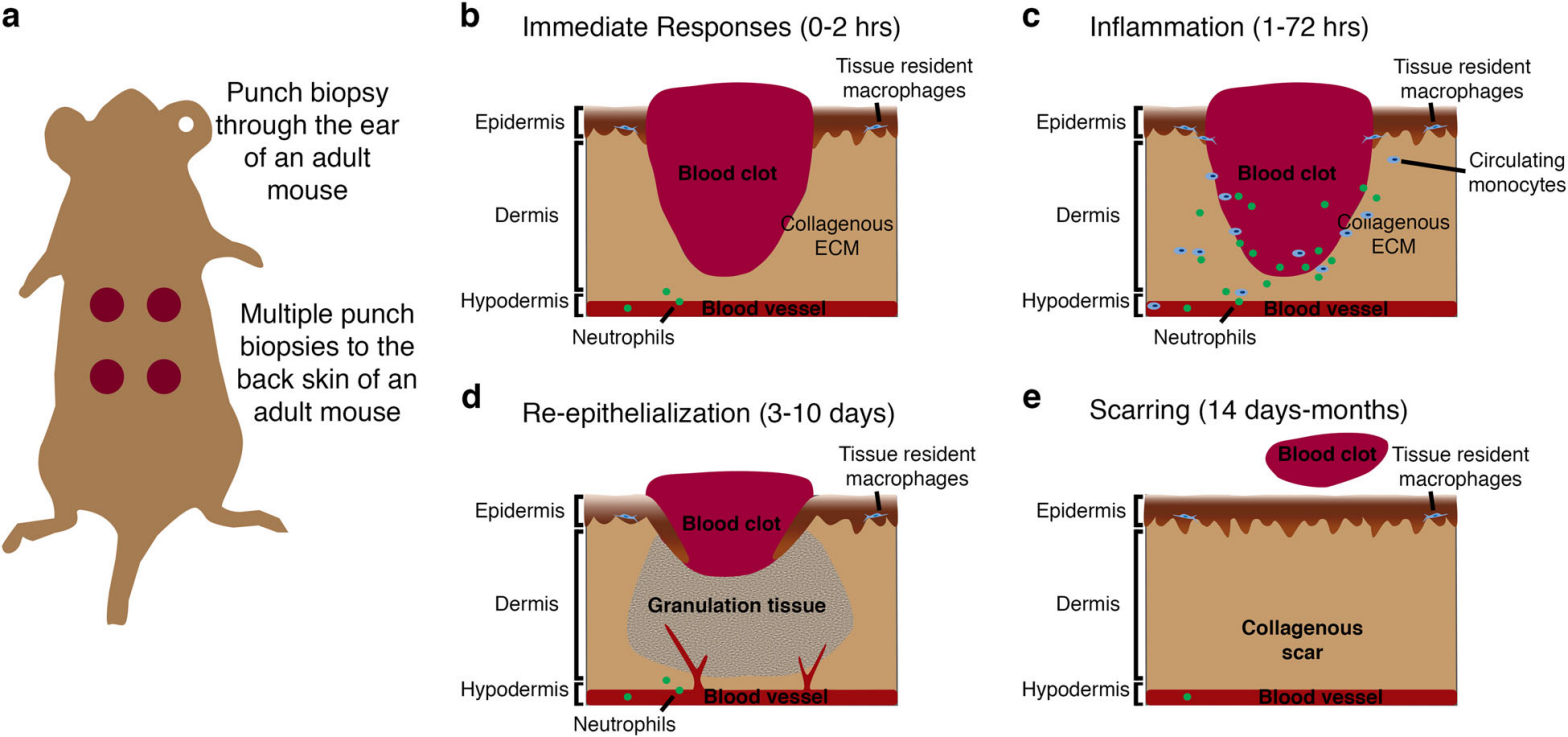
Cutaneous wound healing in adult mouse. **a** Schematic showing method of inducing several (usually 2–4) punch biopsy full-thickness skin wounds to the back skin of a mouse. **b–e** Schematics describing the four main stages of cutaneous healing in adult mouse generally defined as: immediate responses including blood clot formation and neutrophil recruitment (0–2 h; **b**); inflammation involving neutrophil and monocyte recruitment from the peripheral circulation and activation of tissue-resident cells (1–72 h; **c**); re-epithelialization where keratinocytes proliferate and migrate to re-cover the wound, also coinciding with fibrotic granulation tissue formation, collagen deposition and angiogenic sprouting (3–10 days; **d**); and finally, the contraction of the wound by myofibroblasts, wound closure, resolution of inflammation and scar remodelling (14 days–months; **e**)

**Fig. 2:**
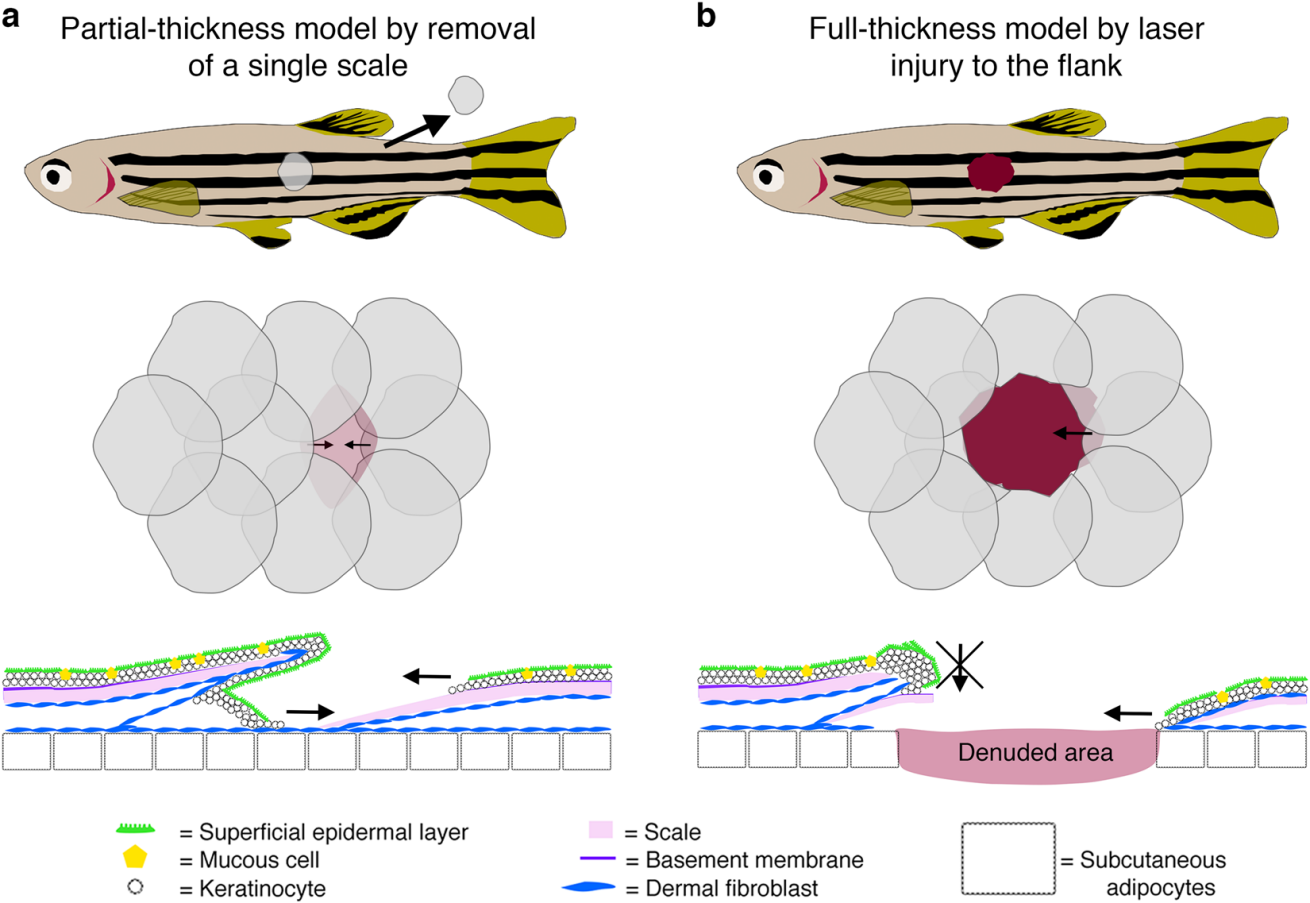
Cutaneous wound healing models in adult zebrafish. **a** Schematic diagrams describing a single scale removal model of partial-thickness cutaneous wounding in adult zebrafish. **b** Schematics describing a full-thickness wounding model in adult zebrafish induced using a dermatology laser as previously described.^6^ In both cases, diagrams at the level of the whole fish (top), flank scales (middle) and cross-section of an individual scale (bottom) are shown. Images were adapted with permission from Richardson et al.^7^

**Fig. 3:**
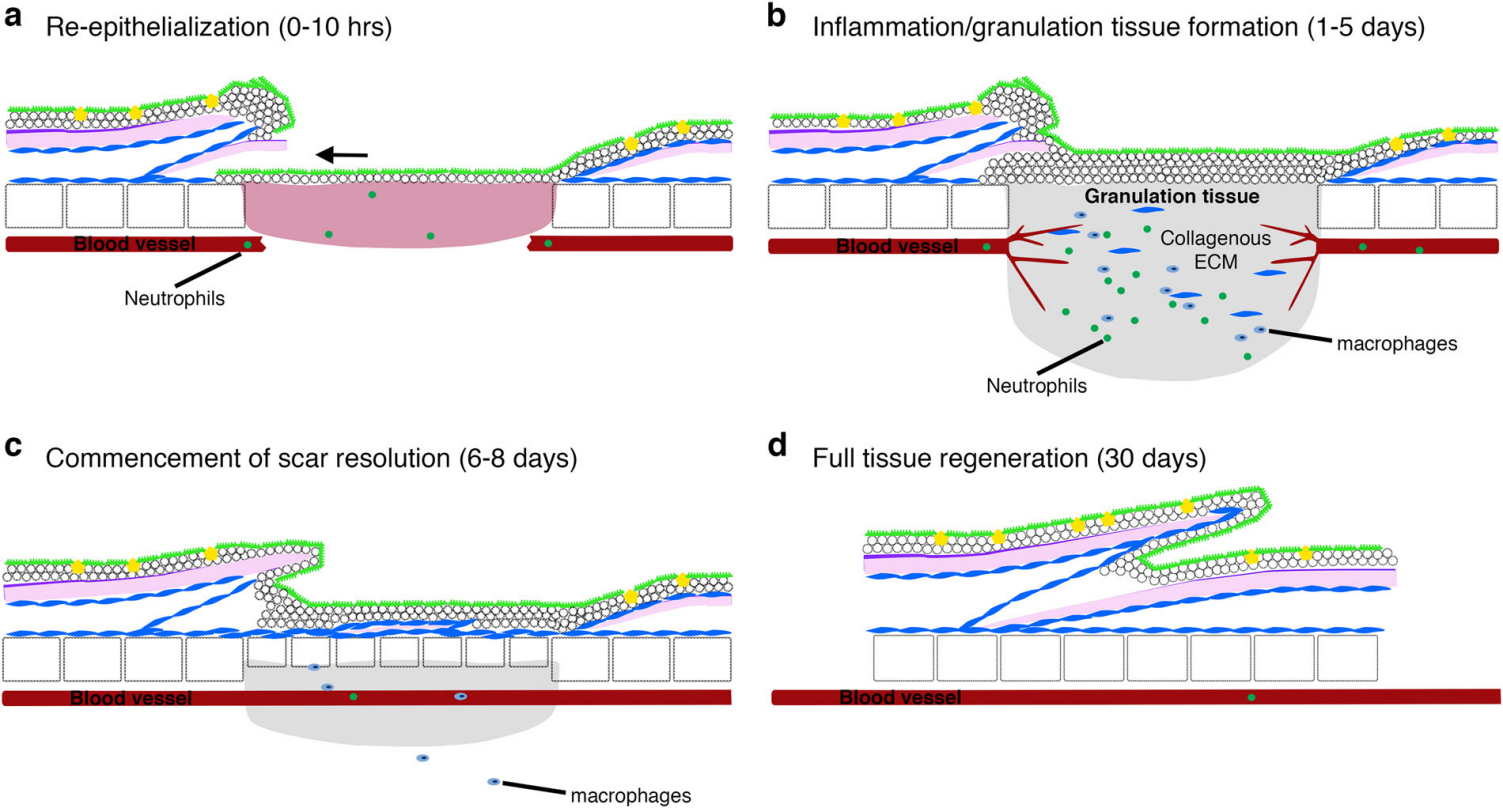
Stages of cutaneous wound healing in adult zebrafish. **a** Schematic diagrams describing the four main stages of cutaneous wound healing in adult zebrafish: the process of re-epithelisation is extremely rapid and completed within 10 h, preceding almost all other cellular responses; **b** once the wound is re-covered, neutrophils and macrophages are recruited, a granulation tissue is formed, neo-vascularisation occurs and collagen is deposited beneath the wound; **c** by 6 days post injury, the granulation tissue and inflammatory responses are reduced and dermal thickenings are starting to reconstitute lost scales; **d** by approximately 30 days after wounding, the tissue is completely regenerated

The skin serves as the primary defence against external pathogen invasion and environmental extremes and regulates body homeostasis via control of the rate of water loss and temperature regulation; therefore, any breach of this primary defence must be rapidly repaired to re-establish this essential barrier function. Differing mechanisms of cutaneous wound healing exist between vertebrate species and between developmental and adult stages and these have been extensively studied allowing the complex interplay of events and cellular interactions that allow for tissue repair to take place to be established (Figs. 1–3). Studies in adult mice, for example, have demonstrated that cutaneous wound healing requires the complex interplay of four main overlapping stages each incorporating different cellular events: immediate injury responses characterised by blood clot formation, inflammatory cell recruitment, re-epithelialization/revascularisation and scar deposition/remodelling (Fig. 1).^10–12^ Due to the non-sterile nature of injuries to the skin, many critical coordinating roles have been suggested for inflammatory cells, which are crucial for fighting external pathogens (described below).

**Fig. 4:**
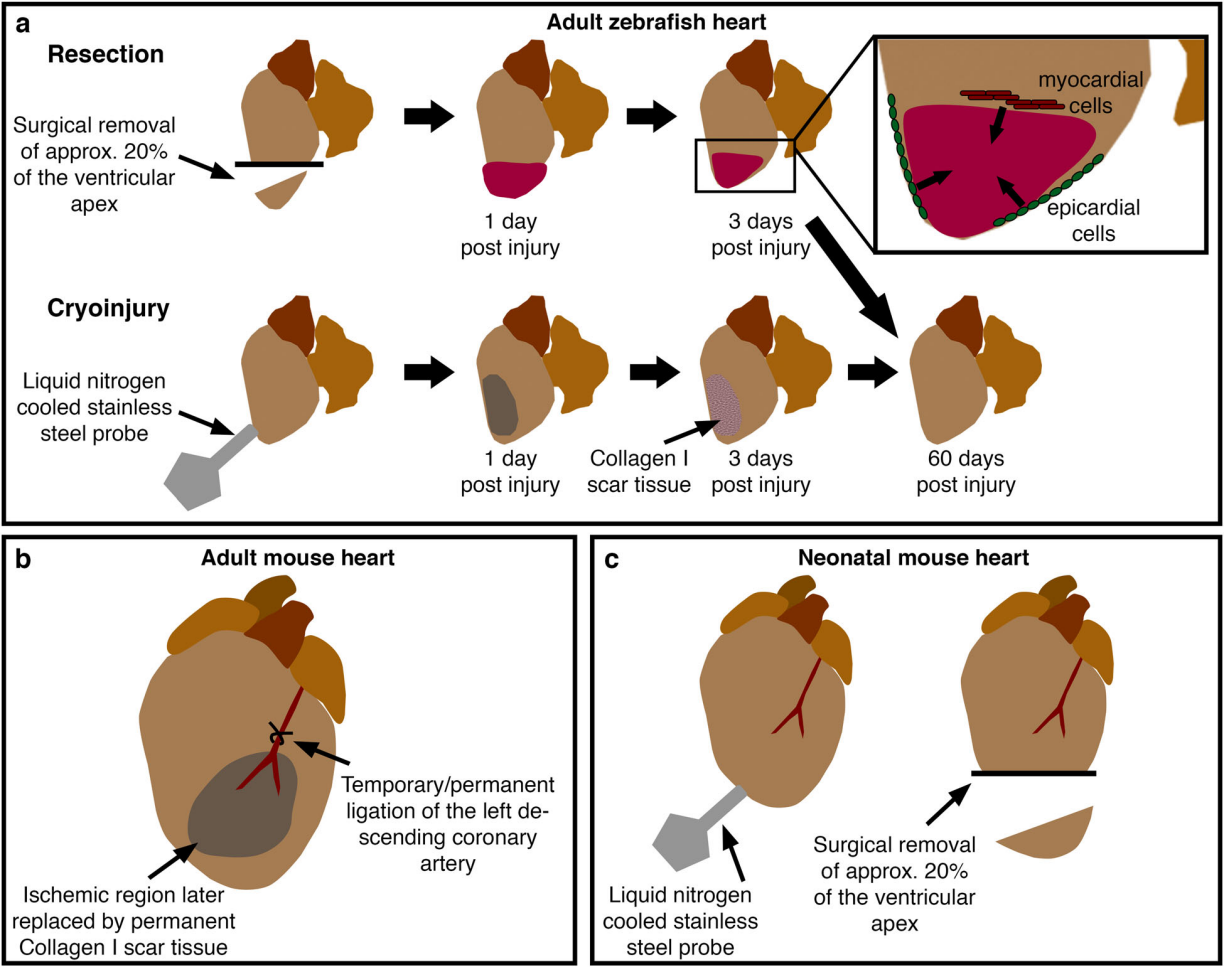
Models of cardiac damage in adult and neonatal vertebrates. **a** Current methods to induce cardiac damage in adult zebrafish include resection, involving the surgical removal of around 20% of the apex of the ventricle, and cryoinjury where a liquid nitrogen-cooled probe is pressed onto the surface of the ventricle, resulting in cell death and transient scarring. These injury models elicit similar modes of healing involving contributions from de-differentiated epicardial and myocardial cells (inset). **b** In adult rodents, cardiac injury is usually induced by ligation of the left descending coronary artery. Temporary ligation allows investigation of the effects of reperfusion injury. **c** Recent reports have described similar injury models to adult zebrafish in neonatal mice eliciting similar cellular responses

By contrast, ischaemic injury to internal organs such as the heart occurs in a sterile environment, but elicits a similar programme of cellular events to that observed following skin injury, triggered primarily by myocardial cell death (Fig. 4). Chronic and acute ischaemic injury in the heart occurs as a result of narrowing/restriction or blockage (myocardial infarction (MI)) of coronary arteries resulting in restricted or absent blood flow and oxygen to a region of ventricular myocardium. MI and chronic ischaemic injury can both occur as a consequence of coronary artery disease and atherosclerosis in humans.^13,14^ The wound healing response to ischaemic injury to internal organs follows a similar programme of cellular events to that of the skin and often results in fibrosis and scarring and a concomitant reduction in tissue function and this process is particularly detrimental to the function of the heart as a mechanical organ. Accordingly, cardiovascular disease remains the biggest killer globally and coronary artery disease accounts for a significant proportion of those deaths.^13,14^ On a positive note, however, advances in surgical interventions, rapid treatment regimens and increased public understanding have vastly reduced the mortality rate for patients suffering an acute MI.^13,15,16^ Despite these advances, the consequences of a severe MI can still include the formation of a permanent scar within the myocardium, limiting the contractility of the ventricle, leading to adverse ventricular remodelling, reducing the capacity to pump blood around the body and increased susceptibility to developing heart failure.^17^ Scar formation within the ventricular myocardium can also lead to progressive degeneration of the surrounding musculature, exacerbating the injury and ultimately leading to an inevitable progression to heart failure. Early heart failure can often be managed with combined medication to reduce blood pressure, relax vessels and reduce heart rate, but more severely affected patients can require more significant treatments including the implantation of a left ventricular assist device, and, for the most severe cases, there is still no curative treatment apart from complete heart transplant. Because of the worldwide prevalence of coronary heart disease and heart failure and the urgent need for improved treatments following extensive cellular damage due to ischaemic injury, therapeutic interventions to replace cardiomyocytes and modify the adverse cellular microenvironment of a scar are under intense investigation and could be of enormous clinical benefit.^16^

Studying the remarkable ability of fish and amphibia to regenerate tissues following traumatic or ischaemic injuries, including replacement of multiple different cell types such as mature cardiomyocytes as well as resolution of interstitial scarring, may hold promise for providing new therapeutic avenues.^4–6,17–20^ In recent years, zebrafish have become an important model organism for studying human disease.^21^ This vertebrate model shares a high degree of genetic homology with humans, they are small, easily maintained, transparent allowing sophisticated live imaging, genetically tractable (allowing the generation of a host of transgenic lines to aid live imaging, as well as forward genetic screens and the rise of CRISPR technology to produce genetic mutants in genes of interest) and are regenerative in multiple organs including the skin and heart allowing detailed cellular level analysis of many disease states. The regenerative capacity of adult zebrafish was first described in the spinal cord and caudal fins with the latter involving cutaneous repair and regeneration alongside other tissues, for example, bone.^22,23^ A thorough understanding of the cellular basis for this regenerative capacity could, therefore, hold the key to improved tissue healing therapeutics of the future.

Multiple different techniques have now been described for inducing skin and cardiac injury in animal models (summary of commonly used techniques shown in Figs. 1, 2 and 4). In adult rodents, full-thickness cutaneous wounds are made by taking several (2–4) punch biopsies of the back skin or through the ear (Fig. 1a).^24,25^ By introducing multiple wounds, several biological replicates can be obtained from the same animal as well as control and test samples.^26^ Similarly, methods for introducing partial (by removal of a single scale) and full-thickness skin injuries to the flank of adult zebrafish using a dermatological laser have been described (Fig. 2).^6^ Several different experimental procedures have also been described to model ischaemic cardiac injury in animals (Fig. 4). In adult mammalian models this is typically performed via ligation of the left anterior descending coronary artery, either permanently or for a fixed period of time and then removed, allowing for the study of reperfusion as well as ischaemic injuries (Fig. 4b). In adult zebrafish, several methods for traumatic and ischaemic cardiac injuries have been described including surgical resection, genetic ablation of cardiomyocytes and the most frequently used, cryoinjury, where a liquid nitrogen-cooled probe is pressed onto the surface of the ventricle to induce an ischaemic injury (Fig. 4a).^4,19,20,27–29^ Similar methods are also used to induce injury to neonatal mouse hearts, which like adult zebrafish hearts retain a capacity for complete regeneration for a limited time period (Fig. 4c).^2,3,30,31^

Here I will discuss the current understanding of the different stages of the injury response comparing cutaneous wound healing and ischaemic injury to the heart as an example of sterile ischaemic injury, paying special attention to recent advances in regenerative models such as the zebrafish.

## Immediate injury responses

In mammals, the immediate response to tissue injury in the skin is platelet activation and commencement of the coagulation cascade, resulting in the formation of a platelet/fibrin-rich clot at the site of blood vessel damage in response to injured endothelial cells (ECs) and other cell types at the site of injury (Fig. 1b).^32^ This fibrin and platelet-rich clot serves as a temporary seal on the injured tissue preventing further blood and fluid loss and reducing invasion by external pathogens.^32^ Platelets embedded within the fibrin clot also release multiple chemotactic and tissue repair-promoting factors that are important for subsequent wound healing processes.^32,33^ In mammalian wound healing, the formation of this fibrin clot precedes the other stages of wound healing and it has been suggested that inhibition of this immediate response affects subsequent healing processes including re-epithelialization.^34–37^ In adult zebrafish, however, no obvious formation of an external fibrin clot is observed (Fig. 2b) following full-thickness skin injury and inhibition of the blood coagulation process has no effect on wound re-epithelialization.^6^ Indeed, wound re-epithelialization is induced extremely rapidly in adult zebrafish and appears to be independent of other wound healing processes, unlike the mammalian situation (see below; Fig. 3a).^6,7^

By comparison, ischaemic injury in the heart does not result in excessive bleeding or the formation of a platelet-rich clot, although endothelial damage can still activate the coagulation cascade resulting in thrombin accumulation and fibrin deposition in the myocardium in rodents and zebrafish.^19,38^ Additionally, ischaemic injury results in activation of the epicardium, endocardium and myocardium and induces the activation of numerous early response genes including *c-fos*, *Hsp70*, *c-jun* and *Erg-1*,^39–42^ many of which are also upregulated in response to cutaneous injury.^43–45^ These early response genes are thought to be protective and induce subsequent crucial wound healing responses, particularly the inflammatory response.^38,41^ Indeed, immune cells respond rapidly to injury signals and very early damage/recruitment signals for inflammatory cells, including hydrogen peroxide and calcium, have recently been identified in zebrafish and Drosophila.^46–48^

In mammalian cutaneous wound healing, the process of re-epithelialization is considered a relatively late phase of the repair response only commencing after a fibrin clot has formed and the subsequent phases of healing have begun (the inflammatory response, granulation tissue formation; see below; Fig. 1d). By contrast, re-epithelialization of large adult zebrafish skin wounds commences immediately after wounding, is completed within hours and is largely independent of all other wound healing phases (Fig. 3a).^7^ Indeed, partial-thickness wounds, induced by removal of a single scale, are re-epithelialized within an hour (Fig. 2a).^7^ This rapid response is facilitated by the more immature form of zebrafish trunk epidermis, consisting only of living cells that are capable of extensive cell elongation and radial intercalation coordinated by Rho/ROCK (Rho-associated kinase) and transforming growth factor-β (TGFβ) signalling.^6,7^ Similarly, following fin resection the surrounding epidermis is mobilised and the epithelial gap is closed within hours.^18^ By contrast, the epithelial layer enveloping the entire heart, the epicardium, is not mobilised to recover the wounded area with epithelium for several days, even in adult zebrafish.^49^ This layer does, however, play several specialist and crucial roles in subsequent cardiac injury responses (see below)

## The inflammatory response

A robust inflammatory response to any tissue injury is an obligate part of the wound healing response. The precise and timely control of this response is arguably the most critical stage required for successful healing with aberrant and extended inflammation being associated with chronic, non-healing skin wounds^50–55^ and reduced inflammation in the heart associated with reduced scarring and cardiac rupture.^56,57^ Multiple different inflammatory cell populations contribute to successful repair in adult mammals and roles for these lineages in complete tissue regeneration in zebrafish and neonatal mice are beginning to be determined.

### Early responders

The commencement of the inflammatory response to tissue injury could be included as an immediate response as circulating leucocytes in the bloodstream and local, tissue-resident populations can be recruited and mobilised within minutes, activated by fast-acting signals including H_2_O_2_ and Ca^2+^.^46,48^ The first inflammatory cells recruited to the site of tissue injury in both the skin and heart are neutrophils of the innate immune system where they perform functions including reactive oxygen species production, release of granular contents and formation of neutrophil extracellular traps, all of which facilitate pathogen killing and subsequent phagocytosis.^58–61^ Neutrophils circulating in the blood rapidly enter the tissue via endothelial attachment and extravasation mechanisms.^62^ Transcriptional profiling of these early responding neutrophils demonstrates that they also play a role in subsequent wound processes including angiogenesis and recruitment of other inflammatory cell types including macrophages and T cells.^60,61,63^ However, knockdown studies suggest that reduction in the number of neutrophils responding to skin wounds has no effect^58^ or even beneficial effects on wounding responses including re-epithelialization, suggesting that these cells can have additional negative effects on the healing response.^64^ Indeed, in adult mammals it has been shown that cutaneous wounds can heal in the absence of any inflammatory response with the main impact restricted to a reduction in scar formation, suggesting a link between these two repair processes.^65^ Similarly, inhibition of the early inflammatory response to cutaneous injury in adult zebrafish does not affect re-epithelialization, the commencement of which precedes all but the earliest immune cell recruitment (Fig. 3a).^6^

Supporting potential predominant negative effects of neutrophils on tissue repair, in the heart studies in adult mice that reduce the number of neutrophils responding to models of MI, demonstrate a reduction in the extent of myocardial injury.^59,66^ This may, in part, be by mediating platelet interactions and the degree of microvascular obstruction, a common problem involving redistribution of neutrophil/platelet plugs to the microcirculation following reperfusion of the major arteries following an MI.^67^ Alternatively, and importantly, however, a recent study has shown adverse effects on cardiac healing following neutrophil ablation,^68^ mediated by alterations in macrophage/monocyte phenotype and recruitment (see below), suggesting additional complexity for the balance of neutrophil function or tissue-specific neutrophil behaviours that have not yet been fully deciphered. Recent reports have also indicated roles for different neutrophil polarisation phenotypes in the response to MI, although the true roles of these different subsets are less well established than for macrophages (see below).^69,70^ Resolution of activated neutrophils is thought to be a critical stage of successful healing. For many years, the dogma has been that neutrophils undergo apoptosis at the site of injury and are cleared by phagocytic macrophages.^71–73^ However, more recent reports, especially using the live imaging advantages of zebrafish, have demonstrated that neutrophils can also survive the early inflammatory phase of an injury and then undergo reverse migration from the injured tissue back into the peripheral blood and that this is an important inflammation resolution mechanism.^74–77^ Further studies will be required to determine the functional and phenotypic effects this reverse migration process has on the surviving neutrophils and zebrafish would be an advantageous model in which to decipher this.^62,76^

Whereas neutrophils exist as a patrolling cell type in the bloodstream and are recruited to the site of injury, other innate immune cell types exist as permanently tissue-resident cells. Macrophages (discussed below) make up the largest proportion of tissue-resident cells in the skin and heart,^78–80^ but other immune cells including mast cells and dendritic cells of the innate immune system and B and T cells of the adaptive immune system are also present in these tissues during homeostasis.^78,80^ These tissue-resident cells can act as early responders as they are already present in the injured tissue and can respond rapidly, although additional cells are often recruited from the peripheral blood (as for macrophages, see below). Precise and important roles are just starting to be assigned for these resident populations. For example, reduced number of activated (degranulating) mast cells is linked to decreased scarring and improved collagen distribution following wounding in mice,^81–84^ partly through reduced accumulation of myofibroblasts.^83,85^ Mast cells are present in regenerative models such as zebrafish,^86^ but their role and the roles of other tissue-resident populations in tissue regeneration has not yet been determined.

### Macrophages

The main innate immune cell type attributed to crucial wound healing functions is the macrophage. A huge array of studies have described a plethora of different macrophage phenotypic states that can have varying roles during homeostasis, tissue repair and disease.^87–89^ Macrophages exist either as tissue-resident cells, both within the skin and heart, or as a population of circulating monocytes in the blood.^90^ Arguably, tissue-resident macrophages could be considered as early responders as they respond to local injury very quickly; however, in adult mammals, it is recruited circulating monocytes that are thought to be the major effectors of downstream tissue injury responses including the induction of scar formation.^91,92^ Indeed, two different origins have been proposed for macrophages, either embryonically derived, which tissue-resident cells are enriched for, or adult bone marrow derived, which mainly contribute to the circulating monocyte population.^91–94^ Resident cardiac macrophages have been shown to be a self-maintaining population via local proliferation with a turnover of approximately 1 month during homeostasis.^91,93^ However, these cells rapidly die or migrate to hematopoietic organs following models of MI^93^ and are then replaced by circulating monocytes, which, again, become self-maintaining once the heart has returned to steady-state after injury.^93^

Studies in neonatal mice, which are also capable of full cardiac regeneration for a limited time period, have recently shown that macrophages are crucial for allowing this regenerative potential.^2,92,95^ These findings extend those of previous findings in spontaneous liver regeneration in rodents.^96^ Macrophages are also required for cardiac and fin regeneration in zebrafish with loss of this cell type resulting in reduced regenerative cell proliferation.^97–100^ Most recently, it has been shown that differences in macrophage expression profiles may contribute to zebrafish regenerative potential.^100^ Interestingly, recent reports suggest that macrophage origin can determine the regenerative capacity of these cells with embryonic-derived macrophages (i.e. tissue-resident) being more anti-inflammatory and pro-angiogenic and so playing more regenerative roles than their adult bone marrow-derived monocyte counterparts, which are more pro-inflammatory in the heart.^92^ This may impact on regenerative ability as inflammation following injury is mediated predominantly by monocytes.^91,92^ However, subsequent findings suggest that this may be too simplistic and that more pro-inflammatory as well as pro-regenerative macrophage subsets may exist and that differentiation of these is governed by their local microenvironment.^89,101^ It is becoming clear that macrophage subtypes have vital and somewhat contradictory roles during tissue repair and that the careful balance of these different phenotypic states will be crucial for allowing full tissue regeneration.^16,51,54,55,87–90,96,100,102–104^ Macrophage subsets have recently been identified in zebrafish^105^ and suggested to contribute to aspects of larval fin regeneration and wound angiogenesis (see section 'Re-vascularisation' below),^106,107^ but further investigation will be required to determine the origin of these different subtypes and if pro-regenerative populations are enriched following skin or cardiac injury in fish, contributing to the regenerative capacity of these organs.

### Late phase responders/inflammation-resolving factors

T cell populations have recently been suggested to play a role in the response to cardiac and cutaneous tissue injury during later stage responses^103,108–113^ and these cells may influence macrophage phenotype.^108,109^ In particular, *Foxp3+* regulatory T cells (T_reg_) have been proposed to promote alternative activation of macrophages post MI and that this process is crucial for correct mammalian cardiac repair.^109^ Multiple different T cell subtypes have been shown to contribute to mammalian tissue injury, but adaptive immunity has been relatively under-studied in regenerative models such as zebrafish. Recent reports have indicated evolutionary conserved roles for Foxp3 as a master regulator of T_reg_ differentiation^114^ and verified the presence of different T cell populations in zebrafish.^115^ Indeed, very recent work has demonstrated a requirement for zebrafish T_reg_ cells in promoting regeneration in a number of organs including the heart via secretion of organ-specific regenerative factors, namely Ntf3 in the spinal cord, Nrg1 in the heart and Igf1 in the retina in a Foxp3-dependent manner.^116^ Further work will be required to fully decipher the cellular differences between mammalian and zebrafish T cells that govern regenerative capacity.

As discussed above, macrophages can exist as a spectrum of different activation states including as anti-inflammatory/pro-resolution mediators. Indeed, studies in zebrafish have shown that macrophages are crucial for regulating inflammatory *interleukin 1 beta* (*il1b*) expression, terminating the early pro-inflammatory response and allowing regenerative blastemal cells to survive following fin amputation.^104^ Changes in macrophage phenotype have been linked to inflammation resolution during tissue injury in mammals,^51,52,55,57,87,90,92,102,109^ but this fails to induce regeneration. The identification of pro-regenerative factors in macrophages of regenerative species such as zebrafish could provide areas of further investigation and therapeutic development.

## Re-vascularisation

Damage signals released at the site of tissue injury also induce the formation of new blood vessels and this is a crucial response to both cutaneous wounds and ischaemic injury to the heart, restoring an oxygenated blood supply and providing nutrients for growth. Multiple growth factors are known to be critical for inducing neo-angiogenesis from existing ECs including vascular endothelial growth factor-A (VEGF-A) and platelet-derived growth factor.^117–119^ In adult zebrafish, rapid re-vascularisation following cryoinjury to the heart is required for cardiac regeneration and VEGF-A is critical for this process.^120^ Indeed, a recent report suggests that rapid administration of synthetic VEGF-A to the ischaemic heart improved cardiac function and long-term survival in a mouse model of MI.^121^ In both adult mammals and adult zebrafish, it is the epicardium of the heart that is thought to provide these pro-angiogenic factors.^117,122^ Indeed, activation of the epicardium has been shown to be crucial for regeneration to take place, dedifferentiating and contributing to new cell types including perivascular cells (see section 'Fibroblasts and scarring', below). Interestingly, a recent report suggests a vital role for pro-inflammatory macrophages in mediating wound angiogenesis in a larval zebrafish wound model via control of anti-angiogenic neutrophils and delivery of VEGF-A,^107^ further supporting the critical role of macrophage subtypes and inflammatory control on disparate aspects of the tissue healing response. Another recent report also suggests a direct role for *vegfaa* in driving adult zebrafish heart regeneration by enhancing cardiomyocyte proliferation as well as inducing neo-vascularisation.^123^

## Innervation

Recent reports have characterised the re-establishment of neuronal networks following cutaneous and cardiac injury and identified crucial roles for this process in driving wound healing and regeneration.^124,125^ In zebrafish and neonatal mouse hearts, cholinergic neurons are required for regeneration following ischaemic and surgical resection of the ventricle with pharmacological and mechanical removal of these neurons resulting in significantly reduced cardiomyocyte proliferation and increased scarring.^124^ In adult mice, lineage tracing identifies peripheral glial-derived cells present in the granulation tissue of cutaneous wounds.^125^ These glial-derived cells are capable of de-differentiation and proliferation and secrete paracrine factors such as TGF-β to induce myofibroblast formation and wound closure.^125^ Studies such as these demonstrate the importance of other cell types in the wound microenvironment for promoting correct cardiac muscle and dermal replacement.

## Fibroblasts and scarring

During normal cutaneous wound healing in mammals, coinciding with re-vascularisation and re-epithelialization, local fibroblasts proliferate and migrate into the region beneath the wound to form the granulation tissue, which will re-establish the dermal connective tissue (Fig. 1d).^10^ Fibroblasts, and subsequent differentiated myofibroblasts, are considered to be the major cell type contributing to the production of extracellular scar material in adult mammals.^10,126,127^ In adult zebrafish, fibroblasts accumulate beneath a full-thickness skin wound to form a granulation tissue similar to what is observed in mammals (Fig. 3b) and which expresses high levels of *col1a1a*.^6^ Similarly, myocardial damage in response to ischaemic cardiac injury and the subsequent inflammatory response (see above) results in the activation of interstitial fibroblasts inducing proliferation, collagen production and differentiation into myofibroblasts in mammals and zebrafish (Fig. 4a, b).^4,128^ Mammals and zebrafish share a similar initial scarring response to tissue injury in the skin and heart. In both situations, a collagen I-rich scar is deposited as a late consequence of the healing process.^4,6^ However, zebrafish gradually remove this scar tissue over time to allow complete tissue regeneration (Figs. 3d, 4a).^4,6^ Recent studies have suggested differences between the collagen secretion profile of fibroblasts present in the zebrafish heart following ischaemic injury at different repair and regeneration stages and demonstrated the importance of this cell type for supporting cardiomyocyte proliferation.^129^

## Regeneration and scar resolution

Full cardiac regeneration in adult zebrafish and neonatal mice relies on the ability of differentiated cardiomyocytes to undergo de-differentiation and to re-enter the cell cycle.^130–133^ This ability is largely lost in adult mammals with current estimates suggesting only a very small percentage of adult cardiomyocytes are capable of cell division,^132,133^ although a recent study suggests that regenerative ability may be variable in adult mammals and this variability may be under genetic control.^134^ Several studies in zebrafish and mice have shown a contribution of the epicardium, the outermost mesothelial layer of the heart, in cardiac regenerative ability. It was initially shown in mice that epicardial cells could be mobilised following ischaemic injury, migrating into the myocardium and contributing de novo cells including cardiomyocytes.^135^ In zebrafish, it has been shown that these cells contribute to cardiac regeneration (Fig. 4a) and it is now thought that epicardial progenitors mainly contribute to non-cardiomyocyte cell types and stem cells supporting re-vascularisation of the injured myocardium, with limited contribution to new muscle cells.^49,136^

In adult mammals, there is limited regenerative response to either acute skin injury or ischaemia and both generally result in the formation of a permanent collagenous scar. Furthermore, full-thickness skin wounds in adult mammals are characterised by a failure in appendage regeneration, for example, hair follicles and sebaceous glands, although a recent study has demonstrated a role for macrophages in inducing hair follicle neogenesis after wounding.^137^ In adult zebrafish, however, regeneration of dermal scales (patterned cutaneous appendages that share some similarities to mammalian hair follicles) occurs after partial or complete ablation during skin wounding (Fig. 3d).^6^ Interestingly, a requirement for cells residing in hair follicles in adult mice and between scales in adult zebrafish has been described to allow for correct and timely re-epithelialisation.^7,138^

Intriguingly, a recent report has demonstrated a regulatory genetic element activated by TGFβ/Activin-β activity, which is transiently activated following both fin resection and cardiac ischaemic injury in adult zebrafish.^139^ This study suggests that a common, genetically controlled programme can contribute to cellular plasticity and regenerative potential in diverse tissues providing the exciting possibility to uncover genetic pathways that could drive regeneration in any affected organ.^139^

## Conclusions

The tissue injury response to acute cutaneous damage or to ischaemic injury to internal organs such as the heart elicit similar programmes of cellular events ultimately leading to fibrosis and scarring in adult mammals. Alternative vertebrate models such as adult zebrafish also exhibit similar injury responses, but are ultimately capable of complete regeneration of these damaged tissue sites. A thorough understanding of the similarities and differences between these regenerative and non-regenerative species can only aid the drive to induce endogenous regenerative potential in adult mammals. Additionally, the identification of common regenerative programmes between tissues and injury types could lead to universal therapeutics in the future, which could drive regeneration in any damaged tissue.
